# Novel Processing Technique to Produce Three Dimensional Polyvinyl Alcohol/Maghemite Nanofiber Scaffold Suitable for Hard Tissues

**DOI:** 10.3390/polym10040353

**Published:** 2018-03-22

**Authors:** Nor Hasrul Akhmal Ngadiman, Noordin Mohd Yusof, Ani Idris, Ehsan Fallahiarezoudar, Denni Kurniawan

**Affiliations:** 1 Faculty of Mechanical Engineering, Universiti Teknologi Malaysia, 81310 Johor Bahru, Malaysia; noordin@fkm.utm.my (N.M.Y.); fallahiarezoudar.ehsan@yahoo.com (E.F.); 2 Faculty of Chemical Engineering, c/o Institute of Bioproduct Development, Universiti Teknologi Malaysia, 81310 Johor Bahru, Malaysia; ani@cheme.utm.my; 3 Department of Mechanical Engineering, Curtin University, 98009 Miri, Malaysia; denni@curtin.edu.my

**Keywords:** electrospinning, 3D printing, thermal inversion phase separation, scaffold, polyvinyl alcohol, maghemite

## Abstract

Fabrication of three dimensional (3D) tissue engineering scaffolds, particularly for hard tissues remains a challenge. Electrospinning has been used to fabricate scaffolds made from polymeric materials which are suitable for hard tissues. The electrospun scaffolds also have structural arrangement that mimics the natural extracellular matrix. However, electrospinning has a limitation in terms of scaffold layer thickness that it can fabricate. Combining electrospinning with other processes is the way forward, and in this proposed technique, the basic shape of the scaffold is obtained by a fused deposition modelling (FDM) three dimensional (3D) printing machine using the partially hydrolysed polyvinyl alcohol (PVA) as the filament material. The 3D printed PVA becomes a template to be placed inside a mould which is then filled with the fully hydrolysed PVA/maghemite (γ-Fe_2_O_3_) solution. After the content in the mould solidified, the mould is opened and the content is freeze dried and immersed in water to dissolve the template. The 3D structure made of PVA/maghemite is then layered by electrospun PVA/maghemite fibers, resulting in 3D tissue engineering scaffold made from PVA/maghemite. The morphology and mechanical properties (strength and stiffness) were analysed and in vitro tests by degradation test and cell penetration were also performed. It was revealed that internally, the 3D scaffold has milli- and microporous structures whilst externally; it has a nanoporous structure as a result of the electrospun layer. The 3D scaffold has a compressive strength of 78.7 ± 0.6 MPa and a Young’s modulus of 1.43 ± 0.82 GPa, which are within the expected range for hard tissue engineering scaffolds. Initial biocompatibility tests on cell penetration revealed that the scaffold can support growth of human fibroblast cells. Overall, the proposed processing technique which combines 3D printing process, thermal inversion phase separation (TIPS) method and electrospinning process has the potential for producing hard tissue engineering 3D scaffolds.

## 1. Introduction

Electrospinning has potential biomedical applications such as in the development of scaffolds [1,2], drug delivery [3,4] and wound dressing [5,6]. In the development of tissue engineering (TE) scaffolds, electrospinning is used due to its simplicity and ability to produce fibers made from many types of polymers at the nanometer scale. The fibers fabricated have submicron diameters with critical structural and instructive component almost replicating the structure of extracellular matrix (ECM) of natural human tissue [7,8,9].

Although electrospinning is a feasible technique for tissue engineering scaffolds, it has its limitation in thickness of the fabricated scaffolds due to the nature of the process [10,11,12]. This means, it is not possible to fabricate a three dimensional (3D) construct using the regular electrospinning process. In order to overcome this limitation, researchers have proposed 3D electrospun tissue engineering scaffolds by making modifications on the electrospinning process. These include redesigning the electrospinning collector [13,14,15], rolling up the nanofiber produced so as to make it multi-layered [16,17], vapor sintering [18], and changing the collector by using a cold plate collector [19,20]. These modifications are able to solve the limitation on the thickness to some extent, but the resulting scaffolds still lack in terms of strength. 

Polyvinyl alcohol (PVA) is a semi-crystalline polymer that possesses good mechanical properties and good chemical and thermal stability [21]. This synthetic polymer is soluble in water, nontoxic, biocompatible, and biodegradable. Therefore, it has the potential among the pool of biomaterials to be used for tissue engineering scaffolds and indeed PVA has been extensively used as the main material to construct tissue engineering scaffolds for hard tissues [22,23,24,25,26,27]. 

Magnetic nanoparticles in the form of maghemite (γ-Fe_2_O_3_) have been used in biomedical applications [28,29,30,31,32] such as cell sheet construction, cell expansion, magnetic cell seeding, cancer hyperthermia treatment, and drug delivery. In our previous work [33], it was reported that the properties of nanofibers mat made from polyvinyl alcohol (PVA)/maghemite (γ-Fe_2_O_3_) nanoparticles exhibited good biocompatibility. Presence of the magnetic nanoparticles within the PVA scaffold also increases its rigidity favourably [34,35] and also enhanced cell growth due to the magnetic field created [33,36]. These magnetic nanoparticles develop a great number of magnetic fields, which would subsequently express osteoinductive effect of static magnetic fields. Each magnetic nanoparticle acts as a single magnetic field and thus when integrated into the matrix, it creates a microenvironment in the pores or on the surface of the blend which sequentially produces the great number of magnetic fields promoting cell proliferation rate. Moreover, maghemite nanoparticles have a large surface area to volume ratio which increases cell area attachment thus allowing more cells to anchor; accommodating a large number of cells [37]. The material characterizations of PVA/maghemite (γ-Fe_2_O_3_) nanoparticles were also discussed in our previous work [33].

Three dimensional (3D) printing is a versatile process which can print objects with any kind of shape and size as required [38]. Recent advances in computational design and higher resolution of the 3D printing process enabled the fabrication of 3D scaffolds with controlled architecture that can mimic natural bone [39,40]. There are several types of 3D printers, including one which works by layering the materials one over another with the supplied materials in filament form. This 3D printing type is called fused deposition modelling (FDM) and it is commonly used due to its simplicity and precision. The limitation of this process is that not all polymeric materials are available in the filament form for use with the FDM 3D printer. Furthermore, the available polymeric materials are in their pure without the addition of any nanoparticles. Specifically, for our study, the PVA filament available is partially hydrolysed thus making it highly soluble in water (dissolves in 10 min) and therefore not suitable to be used directly for fabricating 3D scaffolds. 

The thermally induced phase separation method can result in a scaffold with a microporous structure. Vaquatte and Cooper (2013) [41] developed a 3D scaffold by stacking the thermal inversion phase separation (TIPS) disc with the electrospun disc and they are adhered together by another polymer before freeze drying. After the freeze drying process, a microporous structure was formed on the developed scaffold. 

Considering all of the above, this study attempts to develop a processing technique for fabricating 3D scaffolds made from PVA/maghemite nanoparticles, intended for use in hard tissues. The technique combines 3D printing process, thermal inversion phase separation (TIPS) method, and electrospinning process. As mentioned previously, the FDM 3D printer cannot be used directly to produce tissue engineering 3D scaffold. Instead, the FDM 3D printer is used to produce a good 3D template having the required geometric structure with minute cavities which is part of the moulded structure subjected to the TIPS method. The thermal induced phase separation method can produce the moulded microporous structure of sufficient strength from the 3D printed template and finally electrospinning onto the moulded structure produces nanofibers that can provide the structure that can mimic the extracellular matrix (ECM) structure of natural bone tissue which can enhance the cell growth rate. The fabricated 3D scaffold was then tested for its mechanical properties and biocompatibility.

## 2. Materials and Method

### 2.1. Materials

Chemicals used in this study were reagent grade: iron (II) chloride (FeCl_2_) (98% purity, Sigma Aldrich, Saint Louis, MO, USA), iron (III) chloride (FeCl_3_) (45% purity, Honeywell Riedel-de Haen, Seelze, Germany), sulfuric acid (H_2_SO_4_) (QRëC), nitric acid (HNO_3_) (65% purity, QRëC), ammonia solution (NH_3_) (25% purity, Merck, Kenneth Fort Worth, NJ, USA), hydrochloric acid (HCl) (37% purity, QRëC), fully hydrolysed polyvinyl alcohol (PVA) (99+% purity, with molecular weight 145 kDa, Sigma-Aldrich) and natural PVA filament (partially hydrolysed) for 3D printer.

### 2.2. Novel Processing Technique for Fabricating 3D Tissue Engineering Scaffold

**Figure 1:**
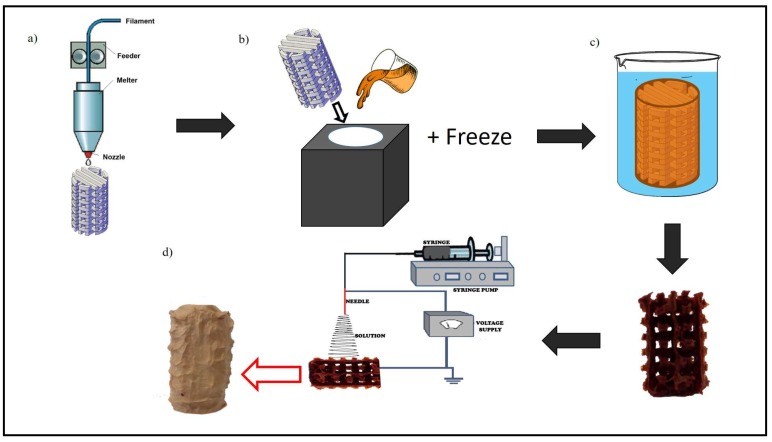
Schematic diagram of the processing technique involved in 3D scaffold fabrication: (**a**) constructing three dimensional (3D) template from partially hydrolysed polyvinyl alcohol (PVA) filament by using 3D printer; (**b**) inserting PVA 3D template into mould and pouring PVA/maghemite solution and freezing it; (**c**) after PVA/maghemite completely solidifies, removing the PVA 3D template by immersing in water; and (**d**) electrospinning PVA/maghemite solution on the 3D PVA/maghemite construct.

The novel processing technique for fabricating 3D tissue engineering scaffolds consist of 3 consecutive steps: (i) initial design of 3D scaffold, printing it using an FDM 3D printer thus forming a 3D template and producing a mould to incorporate the 3D template; (ii) thermally induced phase separation method applied to the moulded structure, and finally; and (iii) electrospinning on surface of the 3D construct. Figure 1 shows the schematic of the processing technique for fabricating the 3D tissue engineering scaffold. 

**Figure 2:**
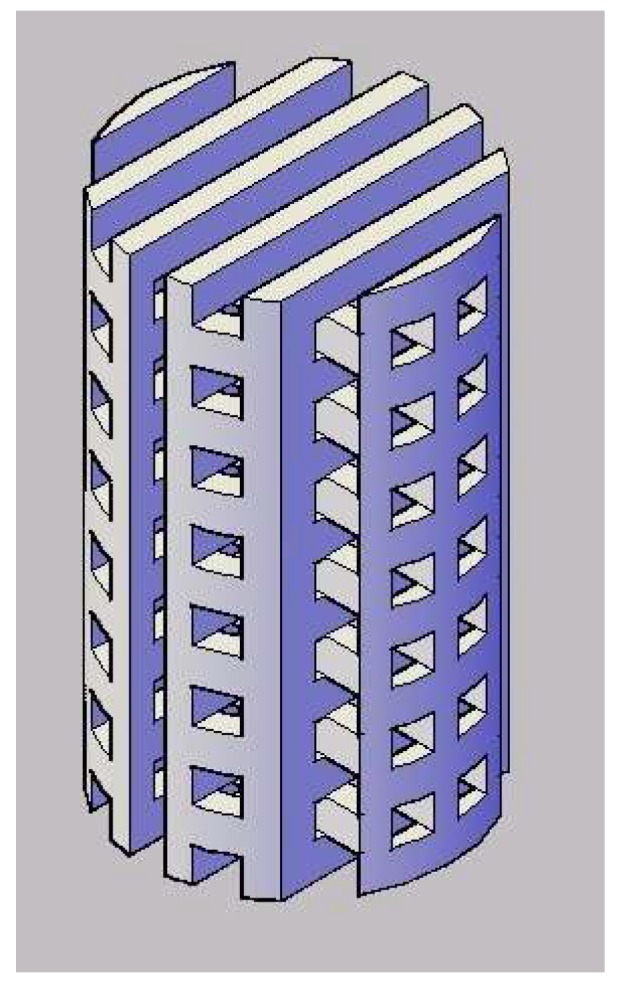
Design of 3D structure.

Initially, the structure with cavities (millipores) sized 2 mm × 2 mm as depicted in Figure 2 was modelled using a computer aided design system and then the 3D template was 3D printed using the FDM process. PVA filament (partially hydrolysed) was used for the FDM process. The extruder temperature was 180 °C and platform temperature was 45 °C. The printing speed used during the process was 80 mm/s.

Upon completion, the PVA 3D printed template (Figure 2) was placed inside a corresponding cylindrical mould. PVA/maghemite solution was then poured into the mould before placing it in a freezer. The diameter and height of the mould is almost similar to that of the template. The prepared PVA/maghemite solution consists of 5% *v*/*v* of γ-Fe_2_O_3_ with 10% *w*/*v* of fully hydrolysed PVA which possesses low degradation rate [42]. After the PVA/maghemite solution has completely solidified in the freezer, the mould was then opened and the solidified structure, after that immersed in liquid nitrogen and then immediately freeze dried for 8–10 h [41] so as to allow further thermal inversion phase separation (TIPS) to occur. This procedure was performed so as to ensure that the PVA/maghemite has a microporous structure and at the same time sufficient strength for hard tissue scaffolds. Finally, it was immersed in distilled water overnight in order to remove the PVA 3D template and the remaining material is a 3D construct made of PVA/maghemite having a negative structure to the original 3D template. After the PVA 3D template was completely dissolved, the PVA/maghemite 3D construct was dried again in an oven at a temperature of 100 °C to remove any residual water. This 3D construct is referred to the 3D construct without wall.

**Figure 3:**
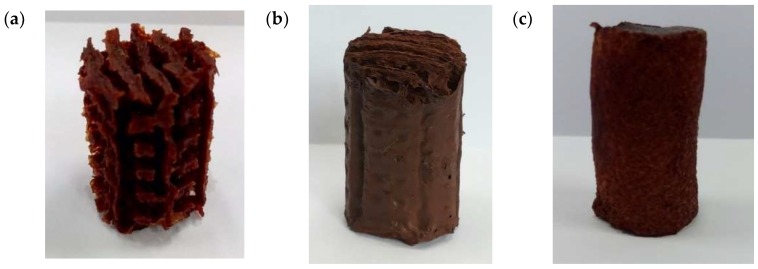
Image of the 3D construct (**a**) without thin wall (**b**) with thin wall (**c**) control specimen.

Another PVA 3D printed template was then produced with a diameter slightly smaller than the initial one. This template was also placed inside the same mould thus creating a thin wall enveloping the template. The purpose of having the thin wall was to provide additional support for the 3D construct. Once the PVA 3D template was ready the same steps above were repeated to produce another PVA/maghemite 3D construct. This 3D construct is referred to the 3D construct with thin wall. The final appearance of both the constructs is shown in Figure 3a,b.

As a control, a solid, cylindrical structure made from PVA/maghemite was also fabricated using the same steps described previously but without a PVA 3D template. The control specimen was also placed in the freezer and then placed in the freeze dryer and electrospun. Without the PVA 3D template, the control specimen has no internal milliporous/cavities structure as shown in Figure 3c. This 3D construct is referred to the 3D construct control specimen.

**Figure 4:**
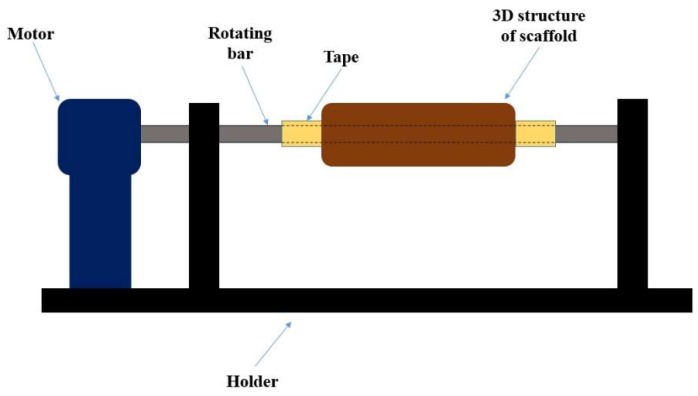
Schematic diagram of rotating electrospinning collector setup.

The above 3D constructs were placed in the electrospinning machine to function as a collector and PVA/maghemite solution was electrospun onto the entire surface of the 3D constructs (Figure 4) so as to form the ECM structure. The PVA/maghemite solution which consists of 7% *v*/*v* nanoparticle content with 10% *w*/*v* PVA concentration was electrospun onto the 3D constructs. The voltage power supply was set at 35 kV with a flow rate of 2.0 mL/h and the distance from the tips to the collector was 80 mm. The rotating speed of the collector was set at 3026 rpm. The speed was increased gradually until it achieved the setting speed, in order to ensure the electrospun nanofibers also penetrated inside the millipores/cavity of the 3D construct when these are present. After the electrospinning process, the constructs were dried under vacuum in order to remove any residual solvent.

### 2.3. Morphology Observation and Mechanical Properties Testing of the 3D Scaffolds

The morphology of the developed 3D PVA/γ-Fe_2_O_3_ scaffolds was examined using field emission scanning electron microscope (FE-SEM) JEOL JSM-7500F (JEOL (M), Petaling Jaya, Malaysia). The cross section image of the scaffolds was obtained by immersing it in liquid nitrogen for 10 min before cutting it using a razor blade. The scaffold was gold coated before examination under the FE-SEM.

For mechanical properties testing, compression test was performed using EZ20KN LLOYD-20 KN universal testing machine (Lloyd, LRX, Singapore). The scaffold was subjected to a compressive load along the axial direction with a 5 kN load cell at a 1 mm/min constant cross head speed. Five 3D scaffolds were tested to ensure reproducibility of data [38]. Statistical analysis using hypothesis testing (*t* test) was performed to test the difference between the mechanical properties of the control and wall 3D scaffolds as well as between the wall and without wall 3D scaffolds. A value of *p* < 0.05 was considered statistically significant.

### 2.4. Initial Biocompatibility Studies on the 3D Scaffolds

#### 2.4.1. In Vitro Degradation Test

The fabricated 3D scaffolds were cut into small square pieces (10 × 10 × 10) mm^3^. The samples were then weighted before placing them in test tubes which contained 30 mL of phosphate-buffered saline (PBS) of pH 7. Each test tube contained 1 sample. The tubes were then immersed in a water bath at a temperature of 37 °C for seven weeks. At different time intervals, samples were taken out from the tubes for evaluation. The degraded samples were rinsed with distilled water and dried at room temperature before weighing. The dried sample’s weight after degradation was recorded. The weight loss was calculated by using Equation (1). The measurement was repeated five times to ensure reproducibility of data [43,44,45,46].

(1) $$\mathrm{Weight}\;\mathrm{loss}=\frac{\mathrm{weight}\;\mathrm{before}-\mathrm{weight}\;\mathrm{after}}{\mathrm{weight}\;\mathrm{before}}\;\times \;100\%$$

#### 2.4.2. Cell Penetration

The cell viability was assessed previously [33]. The result indicates that the presence of maghemite in PVA has increased the cell proliferation rate. For 3D scaffolds, the cell penetration need to be assessed. The fabricated 3D PVA/maghemite scaffolds were sterilized using UV light. Then they were cut into 10 mm height before placing them into a 6-well tissue culture plates. Human skin fibroblast cells (HSF1184) were seeded at a density of 3 × 10^5^ cells/well. The plates were maintained in a humidified atmosphere with 5% of CO_2_ at 37 °C and cultured in DMEM supplemented with 10% FBS, which was changed every 2–3 days. 

The cell morphology inside the scaffolds was examined by using FE-SEM (JEOL JSM-7500F, (JEOL (M), Petaling Jaya, Malaysia). The scaffolds were taken out from the medium, cut into halves, and dried in an oven. The scaffolds were coated with gold and then examined to investigate the cell penetration inside the scaffolds. This procedure was performed on day 5 and 7 of culturing.

## 3. Results and Discussion

### 3.1. Morphology of the 3D Scaffolds

**Figure 5:**
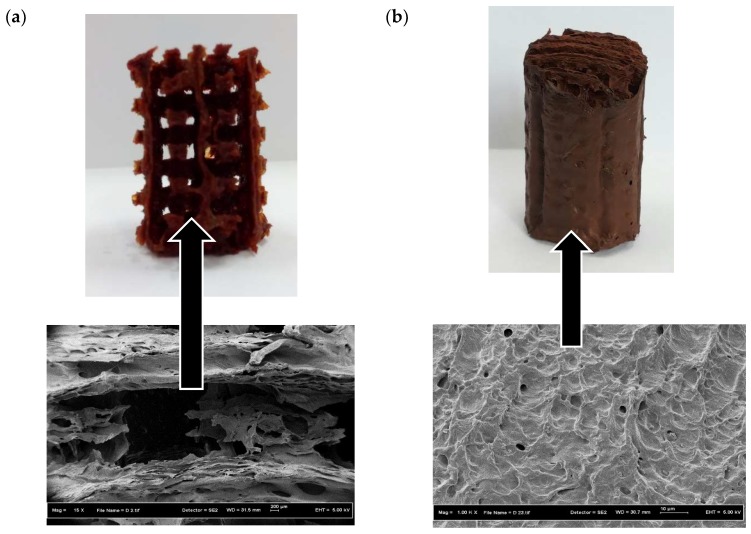

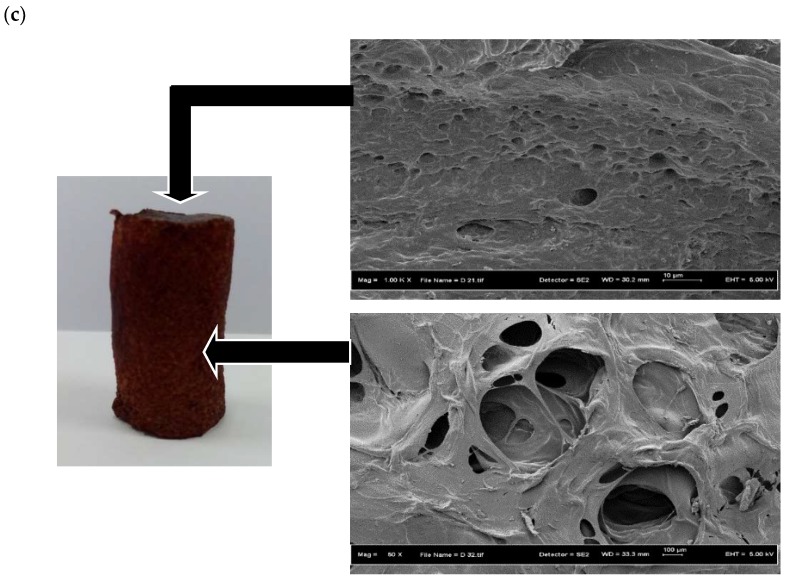
Morphology of the 3D construct: (**a**) without wall; (**b**) with thin wall; and (**c**) control specimen prior to electrospinning.

Figure 5 shows the FE-SEM images of the 3D constructs prior to electrospinning. It is observed that the scaffolds have microporous structure resulted from the freeze drying process. The presence of micropores is expected because they are the sites where cells can attach to and thus enhancing the biocompatibility.

**Figure 6:**
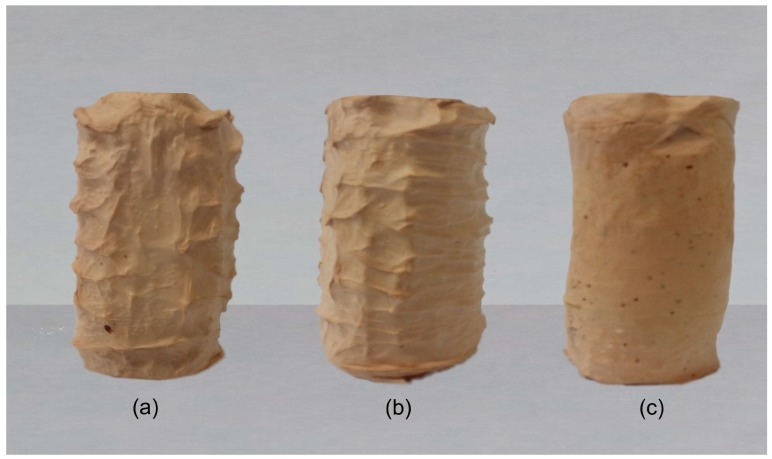
Image of the 3D scaffold: (**a**) without wall; (**b**) with thin wall; and (**c**) control specimen after electrospinning.

The 3D constructs were then used as collectors for the electrospinning process. The 3D constructs were covered by layers of PVA/maghemite electrospun nanofiber and Figure 6 shows the images of the 3D PVA/maghemite scaffold after the electrospinning process. The process parameters setting used during the electrospinning process was the optimal setting based on our previous work [27] which produced nanofibers mats of porosity 90.85% and the thickness of the fibers which covered the entire surface of scaffold was 0.835 mm.

### 3.2. Mechanical Properties of the 3D Scaffolds

**Figure 7:**
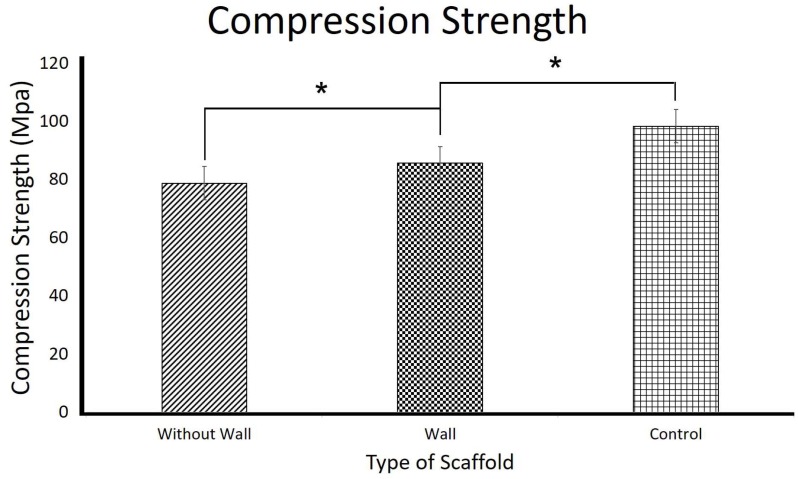
Compressive strength of the 3D scaffolds. (* *p* < 0.05).

**Figure 8:**
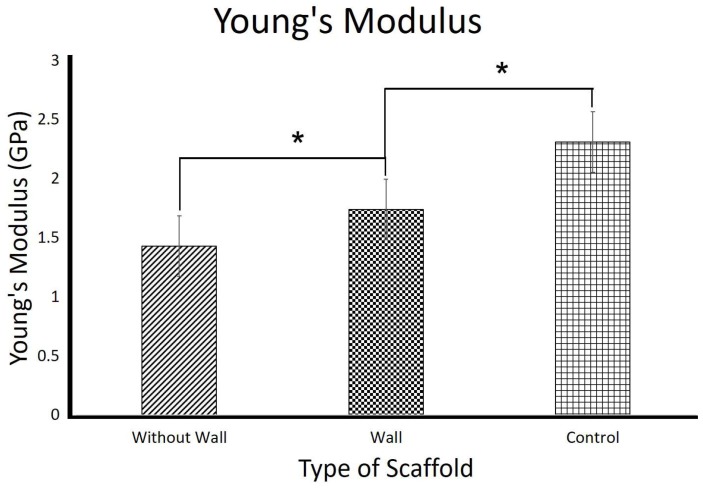
Young’s modulus of the 3D scaffolds. (* *p* < 0.05).

Figure 7 and Figure 8 show the compressive strength and the Young’s Modulus of the 3D PVA/maghemite scaffolds, respectively. Compressive strength of the without wall 3D scaffold is 78.7 ± 0.6 MPa and its Young’s modulus is 1.43 ± 0.82 GPa. These values were much higher than the theoretical values [20] and other 3D scaffolds that were developed using the combined methods which involved electrospinning process and the combining methods which involve electrospinning process for developing 3D scaffold. Previous study, reported a compression strength is 6.69 MPa [18], and Young Modulus are 134.5 MPa [13], 183.57 MPa [47], and 288.05 MPa [48].

From this study, the with wall 3D scaffold mechanical properties (85.6 ± 0.34 MPa compressive strength and 1.74 ± 0.17 GPa Young’s modulus) are slightly higher when compared to the without wall 3D scaffold. The control specimen mechanical properties (98.3 ± 0.61 MPa compressive strength and 2.31 ± 0.47 GPa Young’s modulus) are even higher compared to the with and without wall 3D scaffolds. These trends are related to the porosity of the 3D scaffolds. As reported in literatures, the higher the porosity, the lower the strength and stiffness of the scaffolds [12]. The without wall 3D scaffold has slightly higher porosity compared to the with wall 3D scaffold and the control specimen which is almost solid has much lower porosity compared to the other two 3D scaffolds. The result also indicates that the idea of providing additional support on the scaffold surrounding has not much effect in terms of mechanical properties of the 3D scaffold. The results also indicate that the mechanical properties of the different 3D scaffolds compared were significantly different.

### 3.3. Biocompatibility of the 3D Scaffolds

**Figure 9:**
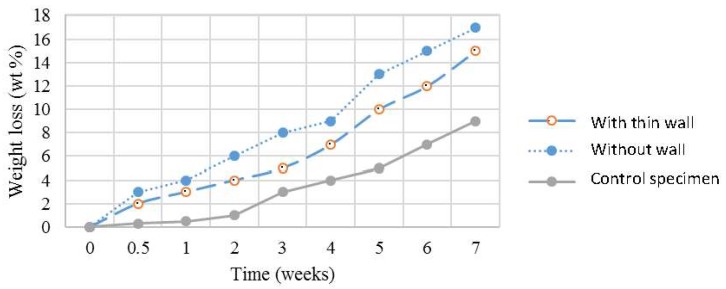
Degradation profile of the 3D scaffolds.

Figure 9 shows the in vitro degradation profile of the 3D scaffolds under simulated body fluid environment. It can be observed that the degradation of the 3D scaffolds happens almost linearly. The 3D scaffold without wall showed the highest degradation rate, followed by the 3D scaffold with wall, while the control specimen was the lowest. The trends in degradation rate are related to the porosity of the scaffolds, where the higher the porosity, the higher the degradation. The mechanism of degradation is suggested to be dissolution of the PVA by the saline water. The 3D scaffold without wall, which has the highest porosity, has also higher surface area where water can penetrate. On the opposite side, the control specimen which has the lowest porosity also has the least contact area with water. 

The degradation rate is an important factor to consider when selecting materials for TE scaffolds. If degradation is too fast, it will reduce the mechanical properties of the scaffolds, making the scaffold unable to withstand the load before hard tissues properly develop or the hard tissues cannot be supported properly during growth. On the contrary, when the degradation is too slow, it will disturb the proliferation of the hard tissues which are supposed to fill in the space occupied by the scaffold. So, degradation rate should be in range (fully degrade in 6–9 months) to make it suitable for tissue regeneration.

Cell penetration study on the 3D scaffolds was performed by seeding human skin fibroblast cells for 5 and 7 days. The purpose of this study is to investigate whether the cells can penetrate inside the scaffolds or not. After 5 and 7 days, the 3D scaffolds were taken out and cut at their cross section. Then the scaffolds were examined by using FE-SEM to find existence of cells inside the scaffolds. 

**Figure 10:**
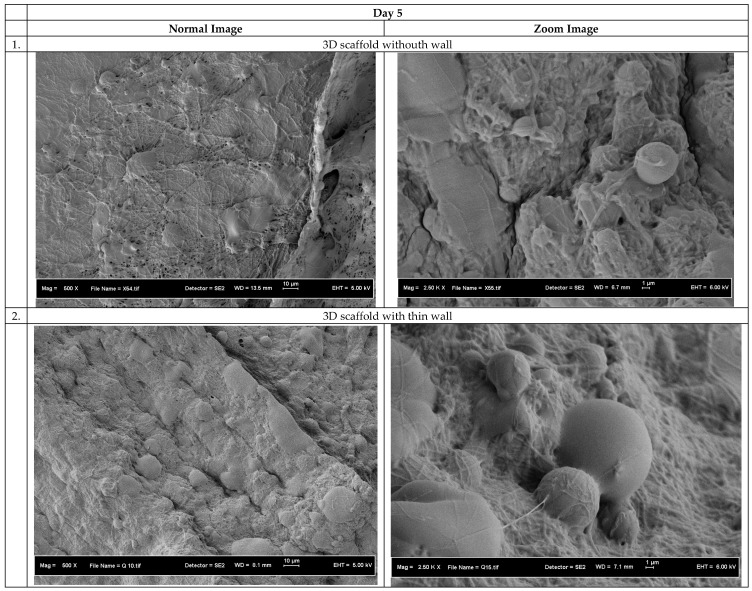

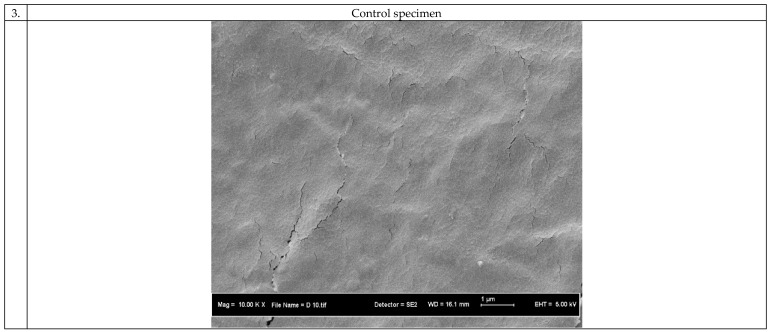
Cell morphology on the cross section of the 3D scaffolds after 5 days’ cell seeding.

**Figure 11:**
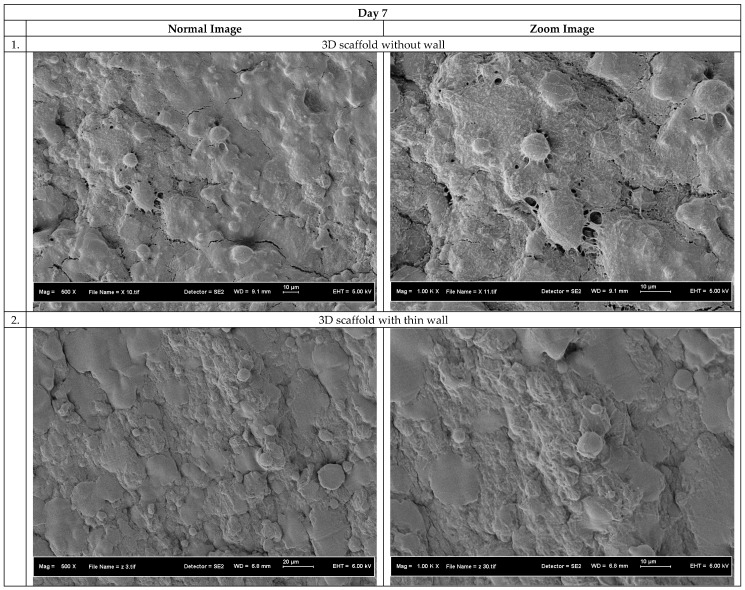

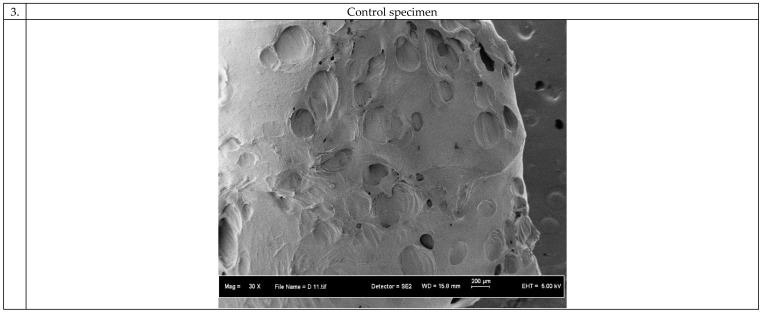
Cell morphology on the cross section of the 3D scaffolds after 7 days’ cell seeding.

Figure 10 and Figure 11 show the FE-SEM images for the cross section of the scaffolds after 5 and 7 days’ cell seeding. The cell penetration was obviously evident on the 3D scaffold without wall and the 3D scaffold with thin wall. From the figure it clearly shows the existence of nanofibers inside the scaffold and cells grow on it. However, there are no nanofibers inside the control specimen due to no millipores on the structure and therefore no cell penetration was observed on the control specimen. The 3D scaffolds without and with thin wall have milli- and micropores structures internally and existence of nanopores externally seem to facilitate cell growth as expected. The high porosity is beneficial for the cells to attach to and to facilitate mass transfer of large amount of tissue liquid for the supply of nutrients to the attached cells, allowing nutrition/gas exchange [49,50,51].

In the case of the control specimen, no cells could grow inside the scaffolds after 5 days of cell seeding. This indicates that the micropores obtained due to freeze drying process were not interconnected, in addition to the fact that the almost solid construct does not have sufficient porosity to facilitate cell growth.

## 4. Conclusions

In this study, a novel processing technique combining 3D printing process, TIPS method, and electrospinning process were used to fabricate 3D scaffolds made from PVA/maghemite. The 3D scaffolds without wall showed good mechanical properties (78.7 ± 0.6 MPa compressive strength and 1.43 ± 0.82 GPa Young’s modulus), within range expected for hard tissue engineering scaffolds. The 3D scaffold without wall has milli- and micropores on its internal structure from 3D printing template and freeze drying and micro- and nanopores on its external structure due to the electrospinning. Degradation of the 3D scaffold under PBS solution is linear, and is likely due to dissolution of PVA in water. For in vitro test, cells were found inside the 3D scaffold with and without wall, indicating it facilitates cell growth as intended. Further tests to evaluate the biological performance in detail will be done by using the bone cell such as MG63 cell lines in future. Overall, the processing technique combining the 3D printing process, TIPS method, and electrospinning process is capable of producing 3D TE scaffolds made of PVA/maghemite which can be applied in hard tissues.
